# Metabolic engineering of roseoflavin-overproducing microorganisms

**DOI:** 10.1186/s12934-019-1181-2

**Published:** 2019-08-26

**Authors:** Rodrigo Mora-Lugo, Julian Stegmüller, Matthias Mack

**Affiliations:** Institute for Technical Microbiology, Mannheim University of Applied Sciences, Paul-Wittsack-Str. 10, 68163 Mannheim, Germany

**Keywords:** Roseoflavin, Riboflavin (vitamin B_2_), *Streptomyces davaonensis*, *Bacillus subtilis*, *Corynebacterium glutamicum*

## Abstract

**Background:**

Roseoflavin, a promising broad-spectrum antibiotic, is naturally produced by the bacteria *Streptomyces davaonensis* and *Streptomyces cinnabarinus*. The key enzymes responsible for roseoflavin biosynthesis and the corresponding genes were recently identified. In this study we aimed to enhance roseoflavin production in *S. davaonensis* and to synthesize roseoflavin in the heterologous hosts *Bacillus subtilis* and *Corynebacterium glutamicum* by (over)expression of the roseoflavin biosynthesis genes.

**Results:**

While expression of the roseoflavin biosynthesis genes from *S. davaonensis* was not observed in recombinant strains of *B. subtilis*, overexpression was successful in *C. glutamicum* and *S. davaonensis*. Under the culture conditions tested, a maximum of 1.6 ± 0.2 µM (ca. 0.7 mg/l) and 34.9 ± 5.2 µM (ca. 14 mg/l) roseoflavin was produced with recombinant strains of *C. glutamicum* and *S. davaonensis*, respectively. In *S. davaonensis* the roseoflavin yield was increased by 78%.

**Conclusions:**

The results of this study provide a sound basis for the development of an economical roseoflavin production process.

**Electronic supplementary material:**

The online version of this article (10.1186/s12934-019-1181-2) contains supplementary material, which is available to authorized users.

## Background

Roseoflavin (8-demethyl-8-dimethylamino-riboflavin) is the only known natural riboflavin (vitamin B_2_) analog with antibiotic activity. At present, this popular research chemical is exclusively produced by chemical synthesis [1]. *N*,*N*-Dimethyl-*o*-toluidine is nitrated to 2-dimethylamino-4-nitrotoluene, which is then reduced with hydrazine in the presence of Raney nickel. The resulting compound 2-dimethyl-amino-4-aminotoluene is condensed with d-ribose to a product, which is pressure hydrogenated in the presence of Raney nickel to 2-dimethylamino-4-*o*-ribitylaminotoluene. This product is condensed with violuric acid to roseoflavin. The resulting crude roseoflavin preparation is acetylated and roseoflavin–tetraacetate is recrystallized from methanol. The purified acetate is then hydrolyzed with NaOH to roseoflavin [1]. Chemically synthesized roseoflavin (molar yield approximately 5%) is commercially available only in mg amounts and depending on the supplier, the market price is between 20 and 30 €/mg.

The potential value of roseoflavin as an antimicrobial agent has been highlighted in various studies [2–7]. Roseoflavin inhibits growth of the bacteria *Staphylococcus aureus* (MIC 1.25 µg/ml) [8], *Enterococcus faecalis*, *Streptococcus pyogenes* [7], *Listeria monocytogenes* (MIC 0.5 µg/ml) [5, 9] and of the protozoal parasites *Trypanosoma cruzi*, *Trypanosoma brucei* and *Leishmania mexicana* [10]. Moreover, roseoflavin is used in the food industry to select roseoflavin-resistant strains. These strains contain mutations which lead to an enhanced expression of riboflavin biosynthetic genes and thus to riboflavin overproduction. For example, two roseoflavin-resistant riboflavin-overproducing strains of *Lactobacillus plantarum* were used for the preparation of bread (by means of sourdough fermentation) and pasta (using a prefermentation step) to enhance their riboflavin content [11, 12]. Notably, these strains are not classified as “genetically modified organisms”.

**Fig. 1:**
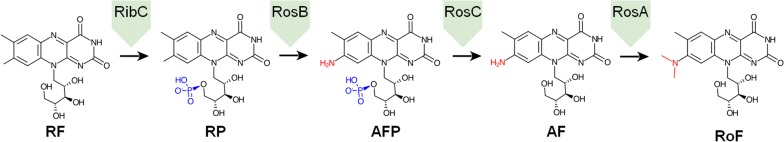
Enzymes RibC (EC 2.7.1.26), RosB (EC 2.6.1.114), RosC (unknown) and RosA (EC 2.1.1.343) responsible for synthesis of roseoflavin (RoF) from riboflavin (RF) via the intermediates RP (riboflavin-5′-phosphate), AFP (8-demethyl-8-amino-riboflavin-5′-phosphate) and AF (8-demethyl-8-amino-riboflavin) [17, 19]. RosA is a dimethyltransferase and first generates the monomethylated form of AF, 8-demethyl-8-methylamino-riboflavin (MAF) (not shown), prior to synthesizing RoF [19]

The Gram-positive bacteria *Streptomyces davaonensis* (formerly known as *Streptomyces davawensis*) and *Streptomyces cinnabarinus* naturally produce roseoflavin and most likely actively secrete it employing a flavin exporter [13–15]. These filamentous bacteria typically grow as cell aggregates which adhere to the surfaces of culture vessels making difficult their culturing and processing of their metabolites. The most important enzymes responsible for biosynthesis of roseoflavin were identified in our group (Fig. 1). Roseoflavin biosynthesis starts with formation of riboflavin-5′-phosphate (RP; also called flavin mononucleotide, FMN) from riboflavin and ATP, a reaction catalyzed by the flavokinase RibC (EC 2.7.1.26) [16]. The complex 8-demethyl-8-amino-riboflavin-5′-phosphate synthase RosB (EC 2.6.1.114) converts RP into 8-demethyl-8-amino-riboflavin-5′-phosphate (AFP; also called 8-demethyl-8-amino-riboflavin mononucleotide, AFMN) which is subsequently dephosphorylated to 8-demethyl-8-amino-riboflavin (AF) by a yet unknown phosphatase (denoted as RosC in Fig. 1) [17, 18]. The dimethyltransferase RosA (EC 2.1.1.343) finally converts AF to roseoflavin via the intermediate 8-demethyl-8-methylamino-riboflavin (MAF) [19].

Very recently the biosynthesis of another flavin analog, coenzyme F_420_ (8-hydroxy-5-deazaflavin) was revised and the new insights now allow the production of F_420_ in a recombinant *Escherichia coli* strain [20].

All bacteria able to catalyze uptake of roseoflavin are likely to be sensitive to this antibiotic [21, 22]. Import of roseoflavin was shown to be mediated by riboflavin transporters [23]. These membrane proteins appear to be widespread in bacteria with eight different riboflavin transporter families described to date [23]. Following uptake, roseoflavin is converted to roseoflavin-5′-phosphate (RoFP or RoFMN) and roseoflavin adenine dinucleotide (RoFAD) by flavokinases (EC 2.7.1.26) and FAD synthetases (EC 2.7.7.2). These enzymes are present in all bacteria whereby many bacteria contain bifunctional flavokinases/FAD synthetases. The latter enzymes were shown to accept riboflavin as well as roseoflavin and other flavin analogs as substrates [9, 16, 24]. The cellular targets for RoFMN are FMN riboswitches and flavoproteins [7, 24, 25]. FMN riboswitches are genetic elements, which control biosynthesis and transport of riboflavin. RoFMN negatively affects FMN riboswitches leading to reduced riboflavin supply and thus to growth reduction of roseoflavin-treated cells [4, 26]. RoFMN and RoFAD both have the potential to interact with flavoproteins and may lead to a reduction of their activity or to their complete inhibition. Since many flavoproteins carry out essential cellular functions [27] their inhibition in turn negatively affects growth. Many bacteria employ FMN riboswitches and all cells depend on the activity of flavoproteins, which is why roseoflavin can be considered a broad-spectrum antibiotic.

Roseoflavin resistance of *S. davaonensis* is at least in part conferred by the membrane protein RibM, which is thought to be responsible for exporting roseoflavin and for importing riboflavin [13]. Moreover, *S. davaonensis* RosA and RosB tightly bind their toxic reaction products roseoflavin and AFP, preventing their interaction with flavoproteins [18]. A RoFMN-insensitive FMN riboswitch controlling expression of riboflavin genes also shown to contribute to roseoflavin resistance of *S. davaonensis* [28].

In this work we enhanced synthesis of roseoflavin in its natural host *S. davaonensis* which as a wild-type produces about 20 µM roseoflavin [14]. In addition, the non-filamentous bacteria *Bacillus subtilis* and *Corynebacterium glutamicum* were engineered to produce roseoflavin. The latter species are popular industrial hosts, which naturally do not synthesize roseoflavin but have been used as hosts to generate riboflavin overproducing strains [29, 30]. Since riboflavin is the precursor to roseoflavin these strains appeared to be well suited for overproduction of this flavin analog.

## Materials and methods

### Chemicals and materials

All chemicals and materials used were of analytical grade and purchased from AppliChem GmbH (Darmstadt, Germany), Carl Roth GmbH & Co. KG (Karlsruhe, Germany) or Thermo Fisher Scientific (Schwerte, Germany), if not otherwise specified.

### Plasmid construction and transformation of bacteria

All plasmids and recombinant bacterial strains used in this study are listed in Table 1 and were constructed according to standard procedures [31]. A synthetic DNA fragment containing the roseoflavin biosynthetic genes *rosB*, *rosA* and the gene *RFK* encoding human riboflavin kinase (RFK) was synthesized by Life Technologies™ (California, USA), and the sequence is shown in Additional file 1: Figure S1 and was deposited in GenBank under the accession number MK541028. All genes were codon-adapted for expression in *B. subtilis*. The synthetic DNA was either used directly for cloning by digestion with specific endonucleases or as a template to generate PCR-products employing oligonucleotides listed in Table 2. The DNA fragments were ligated into different expression vectors by the following restriction sites: pHT01 (*Bam*HI and *Sma*I), pGP888 (*Bam*HI and *Sal*I) and pMKEx2 (*Nco*I and *Kpn*I) to generate the constructs pRML1, pRML2 and pRML3, respectively. For generation of pRML4, the riboflavin transporter gene *ribM* from *S. davaonensis* (extracted from pHT01_ribMopt) (GenBank: FR719838.1) [13] was ligated to *Nco*I-linearized pRML3, using the circular polymerase extension cloning method [32]. Different *B. subtilis* strains (strain 168 and the riboflavin overproducing strain ROP) were transformed with pRML1 and pRML2 to generate the recombinant strains RML1–RML4 using a heat-shock method [33]. *C. glutamicum* was transformed with pRML3 and pRML4 to generate RML5 and RML6 using an electroporation technique [34]. The shuttle vector pSETPermErosArosB [17] containing wild-type *rosA* and *rosB* from *S. davaonensis* (NCBI Gene ID 31229948 and 31229912, respectively) was transferred to *S. davaonensis* by conjugation [17]. The resulting strain was named RML7.

**Table 1:** Plasmids and bacterial strains used in this study

Plasmid or strain	Relevant feature(s)^a^	Source or references
Plasmids		
pMK-RQ-rosB-rosA-RFK	Plasmid containing synthetic (codon-adapted) genes of *rosB*, *rosA* and *RFK* (sequence see Additional file 1: Figure S1); ori_*Ec*_; Kan^R^	Life Technologies™ (California, USA)
pHT01	Expression vector for intracellular production of recombinant proteins in *Bacillus subtilis*. The P_*grac*_ promoter region consists of the *groE* promoter, the *lacO* operator and SD_*gsiB*_. Gene expression is stimulated by addition of IPTG; ori_*Ec*_; Amp^R^; Cm^R^	MoBiTec GmbH (Göttingen, Germany)
pRML1	pHT01 derivative containing *rosB*, *rosA* and *RFK*	This study
pGP888	Expression vector for intracellular production of recombinant proteins in *Bacillus subtilis*; the linearized plasmid integrates into chromosomal *ganA*; gene expression is stimulated by addition of xylose; P_*xylA*_; Kan^R^	[44]
pRML2	pGP888 derivative containing *rosB*, *rosA* and *RFK*	This study
pMKEx2	Expression vector for intracellular production of recombinant proteins in *Escherichia coli* and *Corynebacterium glutamicum* (shuttle vector). Gene expression is stimulated by addition of IPTG; P_T7_, ori_*Ec*_; ori_*Cg*_; Kan^R^	[45]
pRML3	pMKEx2 derivative containing *rosB*, *rosA* and *RFK*	This study
pHT01_ribMopt	pHT01 derivative containing the riboflavin/roseoflavin transporter gene *ribM*; ori_*Ec*_; Amp^R^; Cm^R^	[13]
pRML4	pMKEx2 derivative containing *rosB*, *rosA*, *RFK* and *ribM*	This study
pSET152	Vector for conjugal transfer of DNA from *E. coli* to *Streptomyces*; disrupts *attB* site of *Streptomyces davaonensis* genome; non-replicative in *Streptomyces*; ori_*Ec*_; Apm^R^	[46]
pSETPermErosArosB	pSET152 derivative containing rosA* and rosB* under control of the promoter P_ermE*_	[17]
Strains		
*E. coli*		
Top10	For cloning work	Invitrogen™ (California, USA)
EC01	*E. coli* Top10 containing pHT01; Amp^R^	This study
EC02	*E. coli* Top10 containing pRML1; Amp^R^	This study
EC03	*E. coli* Top10 containing pHT01_ribMopt; Amp^R^	This study
EC04	*E. coli* Top10 containing pGP888; Amp^R^	This study
EC05	*E. coli* Top10 containing pRML2; Amp^R^	This study
EC06	*E. coli* Top10 containing pMKEx2; Kan^R^	This study
EC07	*E. coli* Top10 containing pRML3; Kan^R^	This study
EC08	*E. coli* Top10 containing pRML4; Kan^R^	This study
GM2163/pUB306	Methylation deficient strain used for conjugation with *S. davaonensis*; Str^R^; Cm^R^	[47]
EC09	*E. coli* GM2163/pUB306 containing pSETPermErosArosB; Str^R^; Cm^R^	This study
*B. subtilis*		
WT1	Wild-type strain #1; *B. subtilis* riboflavin-overproducing strain (ROP *aka*. RÜP or BSHP)	[13]
C1	*B. subtilis* ROP containing pHT01 (empty plasmid); control strain; Cm^R^	This study
RML1	*B. subtilis* ROP containing pRML1; IPTG-inducible; Cm^R^	This study
C2	*B. subtilis* ROP containing pGP888 (empty plasmid); control strain; Kan^R^	This study
RML2	*B. subtilis* ROP containing pRML2; xylose-inducible; Kan^R^	This study
WT2	Wild-type strain #2; *B. subtilis* 168 strain	Bacillus Genetic Stock Center (BGSC)
C3	*B. subtilis* 168 containing pHT01 (empty plasmid); control strain; Cm^R^	This study
RML3	*B. subtilis* 168 containing pRML1; IPTG-inducible; Cm^R^	This study
C4	*B. subtilis* 168 containing pGP888 (empty plasmid); control strain; Kan^R^	This study
RML4	*B. subtilis* 168 containing pRML2; xylose-inducible; Kan^R^	This study
*C. glutamicum*		
WT	Wild-type strain; *C. glutamicum* MB001(DE3) strain	[45]
C5–6	*C. glutamicum* MB001(DE3) containing pMKEx2 (empty plasmid); control strain; Kan^R^	This study
RML5	*C. glutamicum* MB001(DE3) containing pRML3; IPTG-inducible; Kan^R^	This study
RML6	*C. glutamicum* MB001(DE3) containing pRML4; IPTG-inducible; Kan^R^	This study
*S. davaonensis*		
WT	Wild-type strain; *S. davaonensis* JCM 4913 strain	Japan Collection of Microorganisms
C7	*S. davaonensis* containing pSET152 (empty plasmid); control strain; Amp^R^; Apm^R^	This study
RML7	*S. davaonensis* containing pSETPermErosArosB; Amp^R^; Apm^R^	This study

^a^P: promoter; SD: ribosomal binding site; ori: origin of replication; Amp^R^: ampicillin resistance; Apm^R^: apramycin resistance; Cm^R^: chloramphenicol resistance; Kan^R^: kanamycin resistance; *rosB*, *rosA*, *RFK* and *ribM*: codon optimized genes for expression in *B. subtilis*; rosA* and rosB*: native genes from *S. davaonensis*

**Table 2:** Oligonucleotides used in this study

Oligo name	Sequence (5′ to 3′)^a^	Restriction sites	Function
RoF_BamHI—Fw	tct aga **gga tcc** gat ggc tct taa agc tct tat cct taa c	*Bam*HI	Vector pRML2 construction
RoF_SalI—Rv	tat gg**g tcg ac**t taa tgg ccg ttc atg att tta gat tta g	*Sal*I	
RoF_NcoI—Fw	cag agc **cca tgg** ctc tta aag ctc tta tcc tta aca caa c	*Nco*I	Vector pRML3 construction
RoF_KpnI—Rv	tcg ag**g gta cc**t taa tgg ccg ttc atg att tta gat tta gaa ac	*Kpn*I	
ribM_NdeI_SpeI—Fw	ttt aac ttt aag aag gag ata ta**c ata tg**a cta gta tga att ggc tga ata gc	*Nde*I	Vector pRML4 construction
ribM_PacI_NcoI—Rv	agg ata aga gct tta aga g**cc atg g**at cct tcc tcc ttt tta att aat tag tgg tgg tga tgg tg	*Nco*I	
pHT01 seq fw	agc gga aaa gaa tga tgt aag cgt	–	PCR and sequence analysis of pRML1
pHT01 seq rev	tcc taa taa gcc gat att agc ctc	–	
lacA 5′—Fw	tat cag ggc ctc gac tac agc	–	PCR and sequence analysis of pRML2
lacA 3′—Rv	cgt ttc aag agg ctc aac tcc	–	
pMKEx2 01—Fw	act cct gca tta gga agc agc	–	PCR and sequence analysis of pRML3 and pRML4
pMKEx2 01—Rv	ttt tag cta tct gtc gca gcg	–	
Pseq1_fw	cga tta agt tgg gta acg cca gg	–	PCR and sequence analysis of pSETPermErosArosB
Pseq7_rev	gca gtg agc gca acg caa tta atg	–	
rosB_dDIG^a^	ctt tgc ctt cga tgt agc cag ggc ctg tgc	–	Northern blot analysis of *C. glutamicum* transformants

^a^Restriction endonuclease sequences are highlighted in bold. The rosB_dDIG probe was ordered with a dual 5′/3′ digoxigenin (DIG) label from Biomers.net GmbH (Ulm/Donau, Germany)

### Template DNA preparation and PCR analysis

Plasmid or chromosomal DNA was isolated from bacterial strains using the “GeneJET™ Plasmid Miniprep Kit” or “GeneJET™ Genomic DNA Purification Kit” from Thermo Fisher Scientific (Schwerte, Germany), respectively. The DNA samples were used as templates for PCR using specific primers (Table 2) to confirm transformation or conjugation of the different bacterial hosts.

### Bacterial growth and processing of fermentation samples

*Bacillus subtilis* strains were aerobically grown in 2xYT (Sigma-Aldrich Chemie GmbH, Munich, Germany) at 37 °C for 18 h (after induction of gene expression) while shaking at 250 rpm. When starting growth experiments cultures were inoculated to an optical density at 600 nm (OD_600_) of 0.15 and, when necessary, gene expression was stimulated at an OD_600_ of 0.8 with 1 mM IPTG (for strains RML1 and RML3) or 2% xylose (for strains RML2 and RML4). *C. glutamicum* strains were aerobically grown in brain heart infusion (BHI) or the minimal medium CGC [35] containing 4% (w/v) glucose at 30 °C for 2–18 h (following IPTG induction) while shaking at 200 rpm. The cultures were inoculated to an OD_600_ of 0.2 and gene expression was stimulated at an OD_600_ of 0.8 with 0.25 mM IPTG. *S. davaonensis* strains were aerobically grown in a yeast-starch (YS) medium [8] at 30 °C for 14 days while shaking at 200 rpm. The media were inoculated to a final amount of 5 × 10^6^ spores/ml. *S. davaonensis* spores were harvested from mannitol soya flour (MS) agar plates. All cultures were prepared independently and in triplicates. Each replicate was used once at a selected incubation day for fermentation sample analyses. If necessary, 5 µg/ml chloramphenicol, 25 µg/ml kanamycin or 50 µg/ml apramycin were added to the growth media. At selected incubation times, samples of the culture supernatants were obtained by centrifugation (10 min, 12,000×*g*, 4 °C), and stored at − 20 °C for HPLC analysis. The remaining cell pellets were used for preparation of cell-free extracts as follows. Bacterial cells were washed twice and suspended in either 50 mM sodium phosphate pH 7.0 (for SDS-PAGE) or a specific buffer for enzyme assays (see “Enzyme assays” section) containing an EDTA-free protease inhibitor cocktail (set III, Merck KgaA, Darmstadt, Germany). Cells were disrupted on ice with 212–300 µm acid-washed glass beads from Sigma-Aldrich and the FastPrep-24™ 5G instrument from MP Biomedicals LLC (California, USA) in 6 cycles at 6.0 m/s. Cell debris and unbroken cells were removed by centrifugation at 15,000×*g*. Cell-free extract samples were immediately used for enzyme assays, or were collected and stored at − 20 °C for HPLC analysis. When necessary, samples were concentrated by evaporation.

### HPLC analysis of flavins

Prior to measuring roseoflavin or other flavins by HPLC, proteins were precipitated from the culture supernatants or cell-free extract samples by mixing with trichloroacetic acid (TCA) to a final concentration of 2.5%. The samples were kept on ice for 5–10 min, centrifuged at 15,000×*g* for 2 min and filtrated through 0.22 µm cellulose-acetate filters. When culture supernatants of *S. davaonensis* were analyzed, TCA treatment was preceded by treatment with α-amylase (Sigma-Aldrich) to degrade starch present in YS. Determination of flavin levels was performed using an Agilent 1260 Infinity system and a 6130 Quadrupole ESI/MS from Agilent Technologies (Waldbronn, Germany) and a biphenyl column (2.6 μm particle size, 150 mm × 2.1 mm) from Phenomenex (Aschaffenburg, Germany). The injection volume was 15 µl and a running buffer containing 35% (v/v) methanol as well as 10 mM ammonium formate (pH 3.7) was used at a flow rate of 0.2 ml/min. Detection of riboflavin, RP, AFP and roseoflavin was carried out using a photometer set to different wavelengths. Riboflavin and RP were detected at 480 nm. AFP and roseoflavin were detected at 480 nm and 509 nm, respectively.

### RNA extraction and Northern blot analysis

Total RNA from bacterial cells was isolated as suggested by the supplier of the “RNeasy Mini Kit” (Qiagen GmbH, Hilden, Germany). RNA samples were transferred to a solid support and hybridized to a specific digoxigenin (DIG)-labelled DNA probe specific for *rosB* (see Table 2) according to standard procedures [31]. Presence of the digoxigenin-labelled probe was determined by chemiluminescence using the “DIG DNA Labeling and Detection” Kit from Roche Diagnostics (Mannheim, Germany).

### Protein determination and SDS-PAGE

Protein was estimated by the method of Bradford using bovine serum albumin as a standard. Proteins were separated under denaturing conditions using polyacrylamide gels (10%) using Mini-PROTEAN^®^ TGX™ Precast Gels from Bio-Rad Laboratories GmbH (Rüdigheim, Germany). Documentation of the resulting gels was accomplished using visible light and staining with Coomassie Brilliant Blue G-250.

### Enzyme assays

Activities of the enzymes RosB, RosA and RFK were determined in cell-free extracts as described earlier [18, 19]. Samples containing 0.5 mg of total cellular protein were added to different assay mixtures. RosB activity was determined at 39 °C in a 100 mM bis–tris-propane (BTP) buffer (pH 8.8) containing 100 µM RP, 10 mM thiamine, 5 mM glutamic acid and 20 µM CaCl_2_. RosA activity was determined at 37 °C in a 50 mM Tris–HCl buffer (pH 8.0) containing 200 µM AF and 2 mM *S*-adenosyl methionine (SAM). RFK activity was determined at 37 °C in a 100 mM potassium phosphate buffer (PPB, pH 7.5) containing 100 µM riboflavin, 1 mM ATP, 6 mM MgCl_2_, 12 mM NaF and 24 mM Na_2_S_2_O_4_. RosB, RosA and RFK were purified as described [17, 18, 36] and used as positive controls. Each reaction sample was mixed with 25 µl of 50% TCA (2.5% final concentration) to stop the reaction. Subsequently, the assay mixtures were prepared for HPLC measurements as described above. The enzymatic activities of RosB, RosA and RFK were confirmed by photometrical detection of AFP, roseoflavin and RP.

### MALDI-TOF MS analyses

Protein bands at the expected molecular mass for the roseoflavin biosynthetic genes products RosB, RosA and RFK were excised from a Coomassie Blue R250-stained SDS-PAGE for in-gel digestion with trypsin. The resulting peptide fragments were analyzed with a Bruker 7 Tesla Fourier transform—ion cyclotron resonance (FT-ICR) mass spectrometer from SolariX-XR, Bruker Daltonics (Bremen, Germany) equipped with a dual ESI MALDI-TOF instrument.

## Results

### Different bacterial hosts show different sensitivities towards roseoflavin

*Bacillus subtilis* wild-type (strain 168) is sensitive to the antibiotic roseoflavin, however, roseoflavin resistant strains easily can be selected on solid growth media [37]. Some of these roseoflavin resistant *B. subtilis* strains were found to overproduce riboflavin which is an antagonist to roseoflavin [37]. A *B. subtilis* wild-type strain (control), the riboflavin-overproducing strain *B. subtilis* ROP and the strain *C. glutamicum* MB001DE3 were tested with regard to roseoflavin resistance to evaluate whether the latter two bacteria potentially could serve as hosts for synthesis of this antibiotic. Different sensitivities towards roseoflavin were observed for these bacteria on solid growth media (Additional file 2: Figure S2). While growth of *B. subtilis* ROP was not affected even at 200 µM roseoflavin, growth inhibition of *B. subtilis* 168 and *C. glutamicum* was observed at 50 µM and 200 µM roseoflavin, respectively. *C. glutamicum* showed strongly reduced growth at 200 µM roseoflavin whereas *B. subtilis* 168 was not able to grow at all, indicating that expression of roseoflavin biosynthetic genes and synthesis of roseoflavin in the cytoplasm of a recombinant *B. subtilis* 168 derivative may be more problematic compared to the other hosts. The natural roseoflavin producer *S. davaonensis* (strain JCM 4913) was previously shown to be roseoflavin-resistant [8, 26] and thus appeared to be well-suited as a potential overproducing production strain.

### Construction of synthetic gene clusters for overexpression of roseoflavin biosynthesis genes

**Fig. 2:**
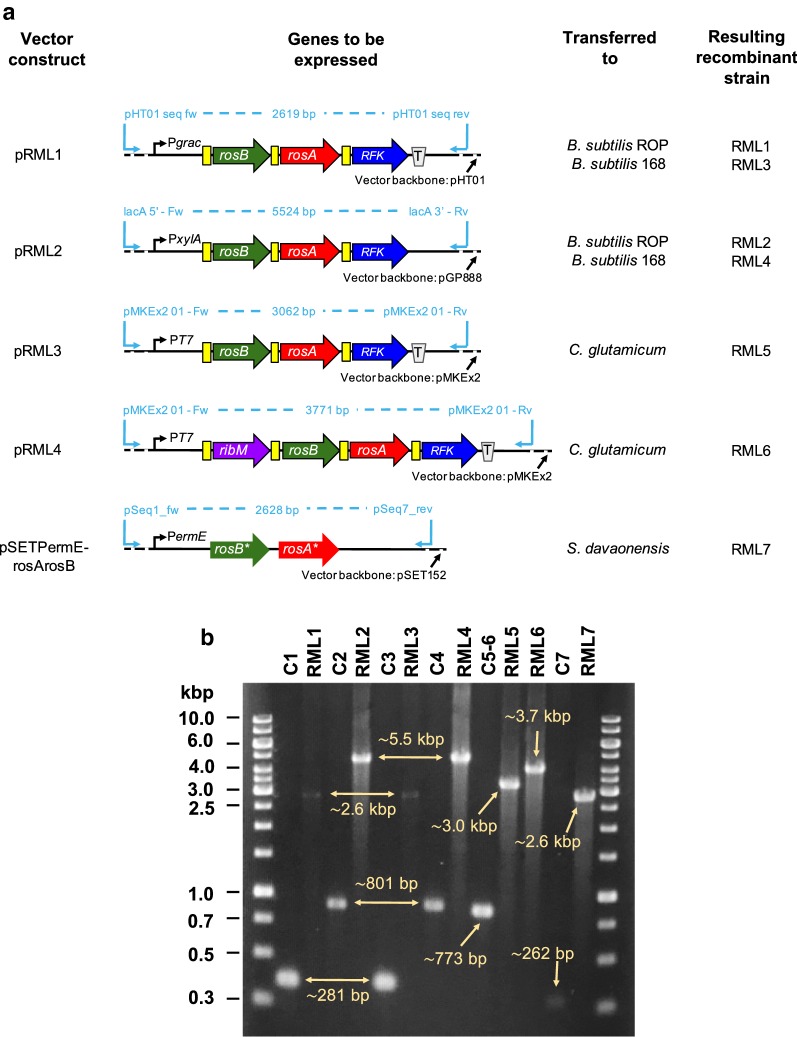
**a** Scheme of expression plasmids used in this study for synthesis of roseoflavin. The genes encoding enzymes for synthesis of roseoflavin (*rosB*, *rosA*), the gene *RFK* encoding a human riboflavin kinase and *ribM* encoding a flavin transporter were arranged as synthetic operons and coupled to different expression plasmids. The resulting vector constructs were introduced into selected bacterial hosts to create the recombinant strains RML1–7. Codon-optimized genes for expression in *Bacillus subtilis* were used in pRML1–4, whereas wild-type genes from *Streptomyces davaonensis* (rosB* and rosA*) were used in pSETPermErosArosB. *P* promoter, *T* terminator; yellow boxes, ribosomal binding sites. Features are not scaled to their original size. **b** PCR analysis of the recombinant bacterial strains used in this study. Amplicons of predicted size (shown by arrows) were produced for each sample using specific primers (for details see Table 2) binding to sequences up- and downstream from the genes to be expressed (RML1–7) or to empty cloning sites (C1–7). Either plasmid DNA (from strains C1, RML1, C3, RML3, C5–6, RML5 and RML6) or genomic/chromosomal DNA samples (from strains C2, RML2, C4, RML4, C7 and RML7) were used as DNA templates

The purpose of the following experiments was to prepare plasmid vectors to allow overexpression of the roseoflavin biosynthetic genes in strains of *B. subtilis*, *C. glutamicum* and *S. davaonensis*. Using commonly used plasmids of each of the species as a basis, five expression vectors were generated as schematically shown in Fig. 2a and transferred to the different bacterial hosts by either transformation or conjugation, to create the recombinant strains RML1–7 (see Table 1). Similarly, empty vectors were introduced into the bacterial hosts to make the control strains C1–7 (see Table 1). In the plasmid constructs, the human riboflavin kinase gene *RFK* was used instead of the endogenous bifunctional flavokinase/FAD synthetase gene *ribC* of *S. davaonensis* to enhance production of the roseoflavin precursor (and substrate for RosB) RP. Contrary to RibC, RFK is a monofunctional enzyme which only produces RP and does not process RP further to FAD [36]. Moreover, the flavin transporter gene *ribM* was included in the construct pRML4 to allow import of riboflavin for synthesis of RP and to allow export of toxic roseoflavin in the production phase. For expression of the genes *rosB, rosA*, *RFK* and *ribM*, both integrative and autonomously replicating plasmids were constructed for *B. subtilis*, whereas only autonomously replicating plasmids and only integrative plasmids were constructed for *C. glutamicum* and *S. davaonensis*, respectively. As PCR analysis validated, all the host cells were successfully transformed or conjugated with the different plasmid constructs (Fig. 2b).

### Expression of the roseoflavin biosynthetic genes *rosB, rosA* and *RFK* was not observed in roseoflavin-resistant *B. subtilis* ROP

In our initial assumptions, *B. subtilis* ROP appeared to be a good host for overexpression of the roseoflavin biosynthetic genes *rosB*, *rosA* or *RFK* because this bacterium overproduces riboflavin (the precursor of RP and consequently of roseoflavin) and is resistant to roseoflavin (Additional file 2: Figure S2). Thus, two different *B. subtilis* ROP recombinant strains RML1 and RML2 were constructed (Table 2) and the roseoflavin biosynthetic genes were either present in the replicative plasmid pRML1 (in RML1) or integrated into the chromosome with pRML2 (in RML2). The inducer compounds IPTG and xylose were added into the liquid growth medium of RML1 and RML2, respectively to drive gene expression. Cell-free extracts of induced cells were prepared and analyzed by SDS-PAGE to assess the presence of the roseoflavin biosynthetic genes products RosB (29 kDa), RosA (38 kDa) and RFK (17.6 kDa). However, in comparison with control strains samples, we could not observe any additional bands on the SDS-PAGE gel, indicating that overexpression of the roseoflavin biosynthetic genes had not occurred (data not shown). Nonetheless, the cell-free extracts of RML1 and RML2, as well as supernatants samples of the corresponding cultures were further analyzed by HPLC to assess for enzymatic activity of RosB, RosA and RFK. Neither roseoflavin nor its precursors AF, MAF, AFP, or RF could be detected by HPLC analyses, either, strongly suggesting that neither of these enzymes were produced in an active form.

Given the apparent inability of *B. subtilis* ROP to express *rosB*, *rosA* and *RFK*, *B. subtilis* 168 was tested as an alternative host. Although, our previous plate assay showed that *B. subtilis* 168 is sensitive to roseoflavin at 50 µM (Additional file 2: Figure S2), the toxic effects of this antibiotic could, in principle, be counterbalanced by adding large amounts of antagonistic riboflavin to the growth medium [37]. Similarly to the *B. subtilis* ROP strains, two new recombinant strains of *B. subtilis* 168 RML3 and RML4 were produced with the plasmid constructs pRML1 and pRML2, respectively, and similarly analyzed. RML3 and RML4 were grown in media supplemented with 400 mg/l riboflavin to ensure high riboflavin levels in the bacterial cytoplasm during gene expression studies. Unfortunately, the recombinant strains of *B. subtilis* 168 RML3 and RML4 were as well unable to overexpress *rosB*, *rosA* and *RFK* and to synthesize active forms of these gene products.

Previously, overexpression of *rosB*, *rosA* or *RFK* in the bacterial host *E. coli* was reported to be possible i.e. prominent additional protein bands corresponding to RosB, RosA or RFK were found [18, 19, 36]. However, only single genes were expressed in these cases. One possible explanation for the lack of overexpression in our recombinant *B. subtilis* strains could be that spontaneous mutations in the foreign gene cassette may have negatively affected its expression or led to inactive roseoflavin biosynthetic enzymes. Therefore, we assessed the presence of mutations of *rosB, rosA* and *RFK* in *B. subtilis* strains by gene-specific PCR and Sanger sequencing of the PCR products from DNA samples isolated at the end of the growth experiments (data not shown). However, sequence analysis showed that none of the sequences was altered, making our hypothesis unlikely. Consequently, we discarded *B. subtilis* (strains ROP and 168) as suitable host for synthesis of roseoflavin. Nevertheless, we propose that Western blotting, Northern blotting or RT-qPCR analysis, which often exhibits great sensitivity, might provide the necessary means to detect, if any, trace expressions of *rosB, rosA* or *RFK* gene products in RML1–4, undetectable in our analyses.

### Expression of *rosB*, *rosA* and *RFK* was observed in *C. glutamicum*

**Fig. 3:**
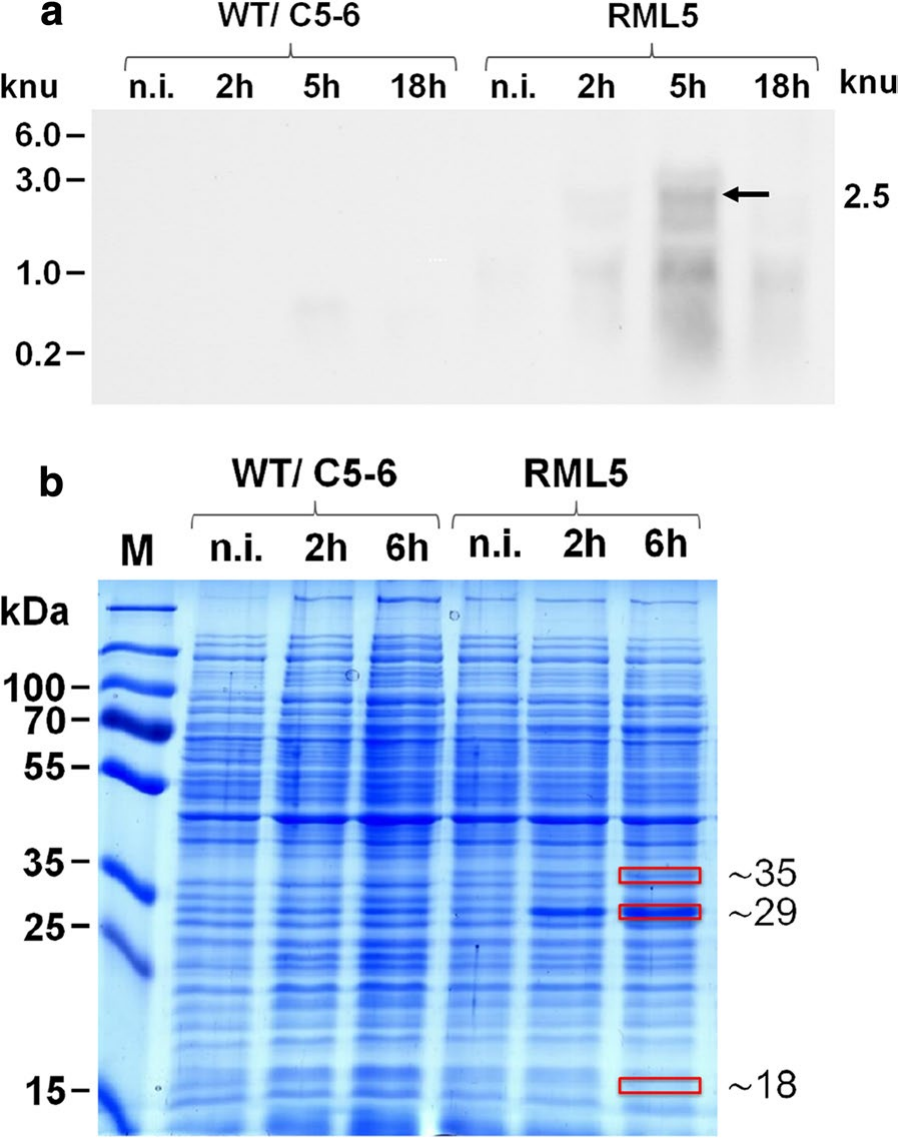
Analysis of gene expression in *Corynebacterium glutamicum*. **a** Northern blot analysis of total RNA isolated from non-induced (n.i.) and IPTG-induced cells of *C. glutamicum* (harvested 2 h, 5 h and 18 h after induction). Total RNA (5 µg each lane) was hybridized to a *rosB*-specific DIG-labelled probe. The expected *rosB*-containing RNA transcript of 2.5 knu is indicated by an arrow. **b** SDS-PAGE analysis of cell-free extracts prepared from non-induced (n.i.) and IPTG-induced cells of *C. glutamicum* strains (harvested 2 h and 6 h after induction). The samples (10 µg of total protein) were loaded onto the gel which after electrophoresis was stained with Coomassie Brilliant Blue R-250. Red boxes indicate the expected molecular masses for the gene products of *rosB* (29 kDa), *rosA* (38 kDa) and *RFK* (17.6 kDa), as well as the excised gel fragments used for MALDI-TOF MS analysis of RosB, RosA and RFK

*Corynebacterium glutamicum* (strain MB001DE3) was evaluated as an alternative host to *B. subtilis* strains for synthesis of roseoflavin. This bacterium was transformed with the replicative plasmid pRML3 containing the roseoflavin biosynthetic genes *rosB, rosA* and *RFK* to make the recombinant strain RML5. Initially, we assessed expression of the roseoflavin biosynthetic genes in this bacterium by Northern Blot analyses (Fig. 3a). Total RNA was isolated from *C. glutamicum* RML5 cultured at different time points after IPTG-induction (2 h, 5 h and 18 h) and hybridized to a *rosB*-specific probe. A maximum expression level of the expected 2.5 knu mRNA (representing all roseoflavin biosynthetic genes) was observed after 5 h of induction in *C. glutamicum* RML5, but not in the controls (Fig. 3a). Shorter transcripts of different molecular sizes in samples of the recombinant strain also hybridized to the *rosB*-specific probe, which were not observed in the *rosB*-lacking parental *C. glutamicum* wild-type strain nor in the control strain C5–6 (Fig. 3a). This indicated that the recombinant mRNA transcripts in RML5 have gone through different stages of RNA degradation. To confirm production of the enzymes RosB, RosA and RFK in *C. glutamicum* RML5 cell-free extracts were analyzed by SDS-PAGE (Fig. 3b). While a strong band at the predicted molecular size for RosB was detected, similarly strong bands corresponding to the expected sizes of RosA and RFK were not found. Nevertheless, gel pieces at the expected molecular sizes for all three proteins were cut out and prepared for MALDI-TOF/MS analysis (Fig. 3b). MALDI-TOF/MS analysis validated the presence of RosB and RosA with a peptide-sequence coverage of 85%, and 67% respectively, but did not confirm the presence of RFK (coverage of just 3%). An explanation for the lower production of RosA and the missing RFK could be the observed instability of *C. glutamicum* RML5-derived mRNA. In all plasmid constructs *rosB* was the first gene immediately downstream of the promoter. It is thus plausible that except for *rosB* translation, translation of the other genes was negatively affected by mRNA degradation (presumably from the 3′-end).

**Fig. 4:**
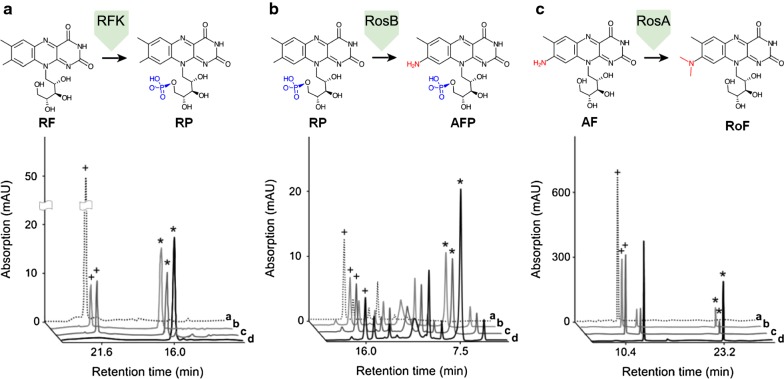
The enzymes RFK, RosB and RosA are active in recombinant *Corynebacterium glutamicum* strains as shown by HPLC analysis of the resulting specific reaction products (structures shown above the graphs). Cell-free extracts of IPTG-induced *Corynebacterium glutamicum* strains (harvested 6 h after induction) were used in different enzyme assays. HPLC-based analysis revealed signals of the assay substrates (marked with a plus +) and the expected reaction products (marked with a star *) for RFK (left graph), RosB (center graph) and RosA (right graph). **a** Control strain C5–6 (negative control), **b** recombinant strain RML5, **c** recombinant strain RML6 and **d** purified recombinant protein (positive control)

*Corynebacterium glutamicum* RML5 was further analyzed by enzyme assays to confirm the presence/absence of roseoflavin biosynthetic genes products RosB, RosA and RFK. Interestingly, we found that when cell-free extracts of RML5 were added to the specific assay substrates RF (RFK activity), RP (RosB activity) and AF (RosA activity), all three expected products RP, AFP and roseoflavin could be detected (Fig. 4). In theory, it could be possible that RF could be converted by another enzyme present in the genome of *C. glutamicum,* which would explain RP formation in the enzyme assay although RFK could not be detected by MALDI-TOF/MS. In fact, *C. glutamicum* naturally encodes an endogenous flavokinase (NCBI Reference Sequence: WP_011014798.1) in its genome which could possibly carry out the same function. However, the cell-free extracts of *C. glutamicum* control strain C5–6 did not lead to RP formation, (Fig. 4), indicating that the substrate conversion was most likely carried out by the recombinant *RFK* in RML5 (although overproduction of RFK was not strong enough to generate a visible band following SDS-PAGE analysis of cell-free extracts). We thus conclude that expression of the biosynthetic genes *rosB*, *rosA* and RFK was successful in our recombinant *C. glutamicum* strain RML5.

**Fig. 5:**
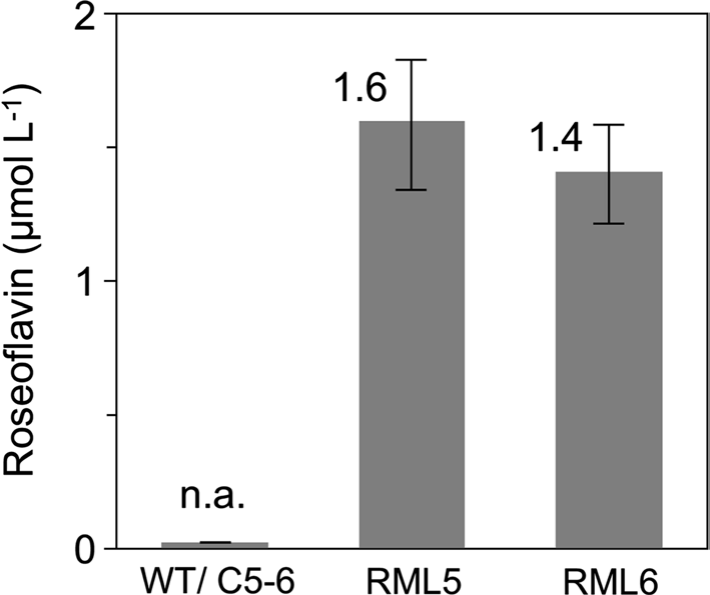
Roseoflavin production in *Corynebacterium glutamicum* strains: parental strain WT (wild-type), control strain C5–6 (containing empty pMKEx2), recombinant strain RML5 (overexpressing roseoflavin biosynthetic genes employing pMKEx2) and RML6 (in addition containing the flavin exporter gene *ribM*). The graph shows the roseoflavin levels present in supernatants of IPTG-induced cells cultivated for 18 h after induction. Each data point represents the average ± SD from cultivations carried out in triplicates

To determine roseoflavin yields in *C. glutamicum* RML5, this bacterium was grown in rich BHI medium to the exponential phase and heterologous gene expression was stimulated by adding IPTG. The color change of BHI during the growth period negatively interfered with the HPLC-based analysis of roseoflavin and thus we were not able to accurately determine roseoflavin levels using our established method. When *C. glutamicum* RML5 was grown in a minimal medium prior to analysis, roseoflavin quantitation was possible. HPLC analysis confirmed that *C. glutamicum* RML5 (and *C. glutamicum* RML6, see following section) was able to produce roseoflavin when grown in minimal medium while the control strains containing empty plasmids did not (Fig. 5). A maximum roseoflavin production of 1.6 ± 0.2 µM (ca. 0.7 mg/l) was measured in IPTG-induced cells of *C. glutamicum* RML5 after 18 h of cultivation.

### The flavin transporter RibM from *S. davaonensis* does not improve roseoflavin yields of a recombinant *C. glutamicum* strain

We observed that cell pellets of *C. glutamicum* RML5 grown in minimal medium to the stationary phase and collected by centrifugation had a pinkish red color, indicating the presence of roseoflavin. We tentatively concluded that at least parts of the roseoflavin remained trapped inside the cells due to the lack of a dedicated roseoflavin exporter and that toxic roseoflavin may slow down central metabolism, preventing the strain from synthesizing larger amounts of roseoflavin. Hence, in a next experiment the *S. davaonensis* gene *ribM* encoding a flavin transporter was co-expressed with the roseoflavin biosynthetic genes *rosB*, *rosA* and *RFK* in strain RML6 (containing pRML4; see scheme in Fig. 2a). The new RML6 and RML5 were similarly cultured in minimal media and expression of the recombinant genes was stimulated by adding IPTG during the exponential phase. At stationary phase (18 h after IPTG-induction) roseoflavin levels were determined in the supernatant of the cultures. Notably, both strains had comparable roseoflavin yields being RML5 even a slightly better producer than RML6 (produced 14% more roseoflavin) (Fig. 5 and Table 3). We measured roseoflavin in the remaining cell pellets of the cultures samples and found out that the intracellular roseoflavin accounted for less than 5% of the total roseoflavin produced in both recombinant strains (nevertheless producing the pinkish red appearance). This suggests that *ribM* does not play a significant role for exporting cytoplasmic roseoflavin in *C. glutamicum*, assuming that a functional RibM was indeed present in the membrane of RML6. Flavins contain a relatively hydrophobic isoalloxazine ring system which explains why roseoflavin (and also riboflavin) have a good potential to diffuse over the cytoplasmic membrane and thus can be found in the supernatants of *C. glutamicum* cultures.

**Table 3:** Fermentation yields of bacterial roseoflavin producers

Bacterial strain	Maximum roseoflavin content (µmol/l)	Cultivation time (day)	Productivity (µmol/l/day)	Relevant yield increase (in %)
*C. glutamicum* RML5	1.6 ± 0.2	1	1.6	14.0 over RML6
*C. glutamicum* RML6	1.4 ± 0.2	1	1.4	na
*S. davaonensis* WT	19.6 ± 2.6	10	1.96	na
*S. davaonensis* RML7	34.9 ± 5.2	10	3.49	78.1 over wt

*na* not applicable

### Expression of additional copies of *rosA* and *rosB* enhance roseoflavin production in a recombinant *S. davaonensis* strain

**Fig. 6:**
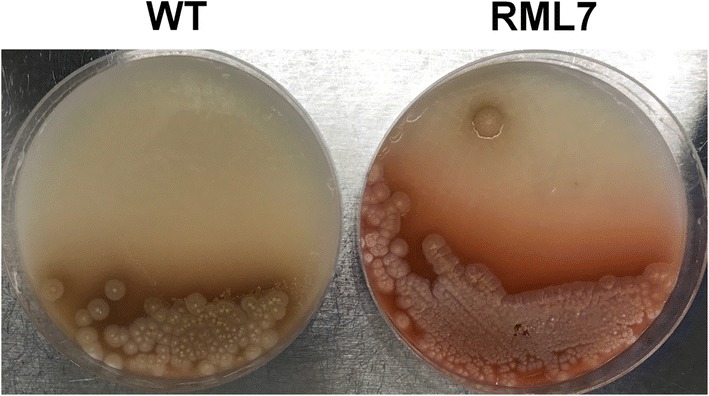
Roseoflavin production of *Streptomyces davaonensis* strains on solid growth media (mannitol soya flour, MS). In contrast to the parental strain WT (wild-type), an extended area of a red, water-soluble pigment is observed around colonies of the recombinant strain RML7, suggesting that roseoflavin overproduction had occurred. An inoculum of 10^5^ spores per MS agar plate was used and the plates were aerobically incubated for 10 days at 30 °C

**Fig. 7:**
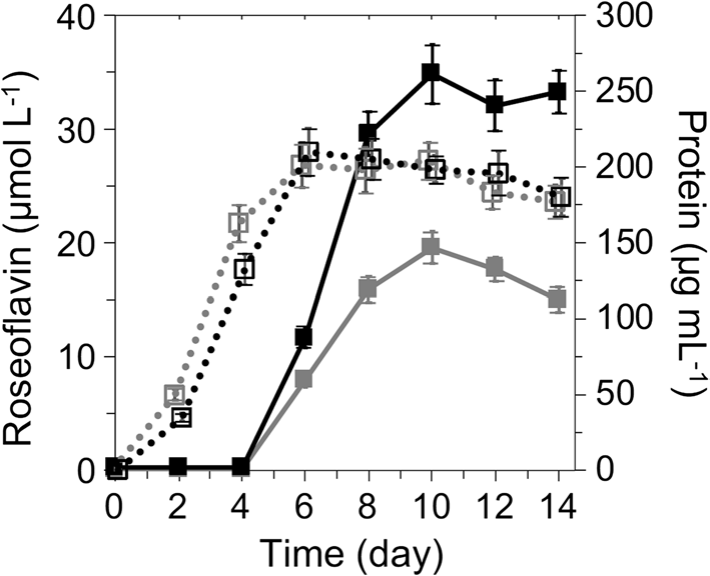
Roseoflavin production by different strains of *Streptomyces davaonensis* in liquid media. Roseoflavin (solid lines) and intracellular soluble protein (dotted lines) contents were measured from culture samples of the parental strain WT (grey squares) and the recombinant strain RML7 (black squares). For each bacterial culture, 5 × 10^6^ spores were used as an inoculum for 300 ml flasks containing 50 ml of yeast starch (YS) medium. The cultures were aerobically incubated at 30 °C for 14 days. Each data point represents the average ± SD from cultivations carried out in triplicates

Additional copies of *rosA* and *rosB* were introduced into *S. davaonensis* by single crossover recombination employing a pSET152 plasmid derivative (pSETPermErosArosB, see Table 1 and Fig. 2), leading to the recombinant strain RML7. The introduced plasmid does not replicate in Streptomycetes but carries the *attP* site and integrase gene of the bacteriophage φC31, enabling it to integrate into the chromosomal φC31 *attB* site in *S. davaonensis*. The plasmid also carries a strong constitutive promoter (P_*ermE*_) to drive expression of wild-type *rosA* and *rosB* and the wild-type ribosomal binding sites of the two genes. Although overproduction of the roseoflavin biosynthetic genes products RosA and RosB in *S. davaonensis* RML7 could not be validated by SDS-PAGE (data not shown), we observed a strongly enhanced roseoflavin production on a solid growth medium (Fig. 6). Fermentation studies employing liquid media were carried out to compare roseoflavin levels in cultures of *S. davaonensis* RML7 and the wild-type strain (Fig. 7). After 10 days of cultivation, maximum roseoflavin productions of 34.9 ± 5.2 µM (ca. 14 mg/l) and 19.6 ± 2.6 µM were measured in *S. davaonensis* RML7 and the wild-type strain, respectively (Table 3). This represents a roseoflavin yield increase of 78% in the recombinant strain. Notably, both *S. davaonensis* wild-type and RML7 strains have similar cell growth, as shown by intracellular soluble protein contents (Fig. 7), indicating that *S. davaonensis* can tolerate roseoflavin overproduction.

## Discussion

With the discovery of the most important enzymes/genes for the biosynthesis of roseoflavin from riboflavin the opportunity to enhance yields of this antibiotic in recombinant strains opened up. Notably, one of the roseoflavin biosynthetic enzymes (an AFP-specific phosphatase) still has not been identified [18] and may represent a bottleneck for roseoflavin production in recombinant strains. In the present study we developed and constructed species-specific plasmids containing the roseoflavin biosynthetic genes *rosB*, *rosA* and *RFK* to synthesize roseoflavin in the non-roseoflavin producers *B. subtilis* and *C. glutamicum* and to overproduce roseoflavin in the natural producer *S. davaonensis*. Initially, we focused on the expression host *B. subtilis* since this bacterium was converted into an excellent riboflavin overproducer by metabolic engineering over the last decades [30, 38] and appeared to be a good host for roseoflavin production for two reasons: First, riboflavin overproduction protects from the toxic effect of roseoflavin and second, the substrate for roseoflavin biosynthesis (riboflavin) is present in large amounts in the cytoplasm of riboflavin production strains such as the *B. subtilis* ROP strain employed in our study. We generated a variety of *B. subtilis* strains where the recombinant genes were either expressed from autonomously replicating plasmids (multiple copies) or from chromosomally integrated plasmids. Although roseoflavin biosynthetic genes were under control of strong, inducible promoter systems we could not verify the expression of any of the recombinant genes in these strains. Mutations to avoid expression of toxic genes were not observed in the sequences of the expression vectors at the end of the growth experiments and we speculate that the reason for absent expression of recombinant genes is the fast phosphorolytic degradation of the recombinant mRNAs which were generated from synthetic genes [39]. These genes were codon-usage optimized for *B. subtilis* but not with regard to mRNA stability. Possibly, introduction of protective secondary structures at the 5′- and 3′-ends would lead to an enhanced mRNA stability and thus expression of *rosA*, *rosB* and *RFK*.

Another popular industrial host, *C. glutamicum*, was successfully employed as a platform to express the roseoflavin biosynthetic genes resulting in roseoflavin synthesis although at comparably low levels (about 1.5 µM). Northern-blot analysis revealed strong degradation of the plasmid-derived synthetic *rosB*-*rosA*-*RFK* mRNA which supports the general idea that synthetic constructs may be more prone to ribonuclease degradation and are thus less stable. We observed that *C. glutamicum* is able to export most of the synthesized roseoflavin (> 95%), even without introducing *ribM* which encodes the *S. davaonensis* flavin transporter [13]. We think that roseoflavin was exported via the homologous flavin transporter PnuX (belongs to the RibM/PnuX-group of transporters [40]) present in *C. glutamicum*, which was previously shown to be able to transport both riboflavin and roseoflavin [41].

RosA only accepts AF as a substrate and not AFP and we are currently trying to identify the missing phosphatase of the roseoflavin pathway. Our previous studies showed that co-expression of *rosA* and *rosB* led to the synthesis of small amounts of roseoflavin in *Streptomyces coelicolor* and in the Gram-negative bacterium *E. coli*, both of which naturally do not synthesize roseoflavin [14, 17, 18]. This indicated that promiscuous phosphatases generated AF in these heterologous hosts and this probably also is the case in *C. glutamicum* as we were able to generate roseoflavin using this expression host.

Judging from our results, the recombinant strain of *S. davaonensis* RML7 appears to be a good choice for roseoflavin production since its productivities almost doubled those of its wild-type and more than doubled those of the recombinant *C. glutamicum* strains (Table 3). Roseoflavin overproduction did not affect growth of *S. davaonensis* which contains a specialized, RoFMN insensitive FMN riboswitch [26], a roseoflavin exporter [13, 16] and the enzymes RosA and RosB which represent a buffer system for toxic riboflavin analogs [18, 19, 28]. In larger industrial settings, however, *C. glutamicum* could be a superior host for roseoflavin production as its cultivation is simpler compared to filamentous *S. davaonensis* [42]. In this study, *C. glutamicum* could reach maximum contents of roseoflavin faster than *S. davaonensis* (1 vs. 10 days, see Table 3) and was capable to secrete roseoflavin. Moreover, there is a significant number of genetic engineering tools available for this bacterium as well as a *C. glutamicum* riboflavin-overproducing strain [43].

## Additional files

**Additional file 1: Figure S1.** Synthetic DNA fragment (2371 bp) synthesized by Life Technologies™ (California, USA). The fragment contains the roseoflavin biosynthetic genes *rosA* (highlighted in green), *rosB* (highlighted in purple) and the human flavokinase gene *RFK* (highlighted in red), which were optimized with regard to the codon-usage of *Bacillus subtilis*. Shine-Dalgarno sequences are highlighted in blue. Unique restriction sites (highlighted in bold) were introduced to the 5′ terminus (*Bam*HI) and 3′ terminus (*Sma*I and *Xho*I).

**Additional file 2: Figure S2.** Roseoflavin (RoF) sensitivity of bacterial strains used in this study. LB agar plates were supplemented with different amounts of roseoflavin (0–200 µM) and inoculated with cells (about 50.000 cells each spot) of *Bacillus subtilis* 168 (A), *Bacillus subtilis* riboflavin overproducing strain ROP (B) and *Corynebacterium glutamicum* MB001DE3 (C) and incubated aerobically for 50 h at 30 °C. *B. subtilis* ROP is able to grow in the presence of 200 µM roseoflavin whereas *B. subtilis* 168 does not show growth. *C. glutamicum* MB001DE3 shows strongly reduced growth (see frames). Please note, the yellowish halos in sectors B are a result of riboflavin secretion by ROP.

## Data Availability

The datasets supporting the conclusions of this article are included within the article.
