# Supplementary material for: Metabolic engineering of roseoflavin-overproducing microorganisms
Source: Microb Cell Fact. 2019 Aug 26;18:146. doi: 10.1186/s12934-019-1181-2 (PMC6709556; doi:10.1186/s12934-019-1181-2)
Supplement: Supplementary file 1 — Additional file 1: Figure S1. Synthetic DNA fragment (2371 bp) synthesized by Life Technologies™ (California, USA). The fragment contains the roseoflavin biosynthetic genes rosA (highlighted in green), rosB (highlighted in purple) and the human flavokinase gene RFK (highlighted in red), which were optimized with regard to the codon-usage of Bacillus subtilis. Shine-Dalgarno sequences are highlighted in blue. Unique restriction sites (highlighted in bold) were introduced to the 5′ terminus (BamHI) and 3′ terminus (SmaI and XhoI). [file 12934_2019_1181_MOESM1_ESM.pdf]

Additional data to the manuscript:

Metabolic engineering of roseoflavin-overproducing microorganisms

Rodrigo Mora-Lugo, Julian Stegmüller and Matthias Mack*

Institute for Technical Microbiology, Mannheim University of Applied Sciences, Paul-Wittsack-Str. 10, 68163 Mannheim, Germany

***Correspondence:** m.mack@hs-mannheim.de

Institute for Technical Microbiology, Mannheim University of Applied Sciences, Paul-Wittsack-Str. 10, 68163 Mannheim, Germany

5'_**GGATCC**ATGGCTCTTAAAGCTCTTATCCTTAACACAACACTTCGTCGTTCTCCTTCTCGTTCTCAAACACAAGGCCT
TATCGATAAAGCTGTTCCTCTTTACGAAAAAGAAGGCATCGAAACAGAAGTTGTTCGTGTTATCGATCATGATATCGAAC
AAGAATACTGGGATGATTACGATGATTGGAACGCTGGCGAAAAAGCTCGTCGTGAAGATGAATGGCCTTGGCTTCTTGAA
AAAATCCGTGAAGCTGATATCCTTGTTATCGCTACACCTATCACACTTAACATGTGCACATCTGCTGCTCATGTTATCCT
TGAAAAACTTAACCTTATGGATGAACTTAACGGCGATACAAAACAATTCCCTCTTTACAACAAAGTTGCTGGCCTTCTTA
TGTGCGGCAACGAAGATGGCGCTCATCATGTTGCTGGCACAGTTCTTAACAACCTTGGCCGTCTTGGCTACTCTGTTCCT
CCTAACGCTGCTGCTTACTGGCTTGGCCCTGCTGGCACAGGCCCTGGCTACATCGAAGGCAAAGGCGATCGTCATTTCCA
TACAAACAAACTTATCCGTTTCATGGTTGCTAACACATCTCATCTTGCTCGTATGCTTCAAGAAACACCTTACACAACAG
ATCTTGAAGCTTGCGCTCAAGCTGCTCGTGAAGAATCTGATGATGTTTTCGCTATCCGTGTTAACGTTAACACACCTGCT
ATCCGTTACAAACGTTTCCAAAAACTTGGCGAAGTTAAAGTTGAAGAATCTCAACTTGGCTAACAATCTAGAGAGCTCTT
AAAGGAGGAAGGATACATGCGTCCTGAACCTACAGAACATCCTGAACGTACAGCTGCTCAACGTCTTTACCAATACAACG
TTGATCTTAAAGTTGCTTTCGTTCTTTACGCTGTTGCTAAACTTCATCTTCCTGATCTTCTTGCTGATGGCCCTCGTACA
ACAGCTGATCTTGCTGCTGCTACAGGCTCTGATCCTTCTCGTCTTCGTCGTCTTCTTCGTGCTGCTGCTGGCGCTGATGC
TCTTCGTGAAGTTCCTGAAGATTCTTTCGAACTTGCTCCTATGGGCGATCTTCTTCGTTCTGGCCATCCTCGTTCTATGC
GTGGCATGACAACATTCTTCGCTGAACCTGATGTTCTTGCTGCTTACGGCGATCTTGTTGAATCTGTTCGTACAGGCGTT
CCTGCTTTCCAACTTCGTCATCGTGAACCTCTTTACGATTTCCTTGCTCGTCCTCAACATAAAGAAGTTCGTGATGAATT
CGATGCTGCTATGGTTGAATTCGGCCAATACTTCGCTGATGATTTCCTTACATCTTTCGATTTCGGCCGTTTCACACGTT
TCGCTGATATCGGCGGCGGCCGTGGCCAATTCCTTGCTGGCGTTCTTACAGCTGTTCCTTCTTCTACAGGCGTTCTTGTT
GATGGCCCTGCTGTTGCTGCTTCTGCTCATAAATTCCTTGCTTCTCAAAACCTTACAGAACGTGTTGAAGTTCGTATCGG
CGATTTCTTCGATGTTCTTCCTACAGGCTGCGATGCTTACGTTCTTCGTGGCGTTCTTGAAGATTGGGCTGATGCTGATG
CTGTTCGTCTTCTTGTTCGTATCCGTCAAGCTATGGGCGATGCTCCTGAAGCTCGTCTTCTTATCCTTGATTCTGTTATC
GGCGAAACAGGCGAACTTGGCAAAGTTCTTGATCTTGATATGCTTGTTCTTGTTGAAGGCGAACATCGTACACGTGCTCA
ATGGGATGATCTTCTTGCTCGTGCTGGCTTCGATATCGTTGGCATCCATCCTGCTGGCGATGTTTGGCTGTTATCGAAT
GCCGTGGCACAGCTGGCTAAGACGTCCAATTGCGGCCGCAAAGGAGGAAGGATAATGCGTCATCTTCCTTACTTCTGCCG
TGGCCAAGTTGTTCGTGGCTTCGGCCGTGGCTCTAAACAACTTGGCATCCCTACAGCTAACTTCCCTGAACAAGTTGTTG
ATAACCTTCCTGCTGATATCTCTACAGGCATCTACTACGGCTGGGCTTCTGTTGGCTCTGGCGATGTTCATAAAATGGTT
GTTTCTATCGGCTGGAACCCTTACTACAAAAACACAAAAAAATCTATGGAAACACATATCATGCATACATTCAAAGAAGA
TTTCTACGGCGAAATCCTTAACGTTGCTATCGTTGGCTACCTTCGTCCTGAAAAAAACTTCGATTCTCTTGAATCTCTTA
TCTCTGCTATCCAAGGCGATATCGAAGAAGCTAAAAAACGTCTTGAACTTCCTGAACATCTTAAAATCAAAGAAGATAAC
TTCTTCCAAGTTTCTAAATCTAAAATCATGAACGGCCATTAA**CCCGGGCTCGAG**_3'

Fig. S1: Synthetic DNA fragment (2371 bp) synthesized by Life Technologies™ (California, USA). The fragment contains the roseoflavin biosynthetic genes *rosA* (highlighted in green), *rosB* (highlighted in purple) and the human flavokinase gene *RFK* (highlighted in red), which were optimized with regard to the codon-usage of *Bacillus subtilis*. Shine-Dalgarno sequences are highlighted in blue. Unique restriction sites (highlighted in bold) were introduced to the 5' terminus (*Bam*HI) and 3' terminus (*Sma*I and *Xho*I). This sequence has been deposited in GenBank under the accession number MK541028.
