# Supplementary material for: Metabolic engineering of roseoflavin-overproducing microorganisms
Source: Microb Cell Fact. 2019 Aug 26;18:146. doi: 10.1186/s12934-019-1181-2 (PMC6709556; doi:10.1186/s12934-019-1181-2)
Supplement: Supplementary file 2 — Additional file 2: Figure S2. Roseoflavin (RoF) sensitivity of bacterial strains used in this study. LB agar plates were supplemented with different amounts of roseoflavin (0–200 µM) and inoculated with cells (about 50.000 cells each spot) of Bacillus subtilis 168 (A), Bacillus subtilis riboflavin overproducing strain ROP (B) and Corynebacterium glutamicum MB001DE3 (C) and incubated aerobically for 50 h at 30 °C. B. subtilis ROP is able to grow in the presence of 200 µM roseoflavin whereas B. subtilis 168 does not show growth. C. glutamicum MB001DE3 shows strongly reduced growth (see frames). Please note, the yellowish halos in sectors B are a result of riboflavin secretion by ROP. [file 12934_2019_1181_MOESM2_ESM.pdf]

Additional data to the manuscript:

Metabolic engineering of roseoflavin-overproducing microorganisms

Rodrigo Mora-Lugo, Julian Stegmüller and Matthias Mack*

Institute for Technical Microbiology, Mannheim University of Applied Sciences, Paul-Wittsack-Str. 10, 68163 Mannheim, Germany

***Correspondence:** m.mack@hs-mannheim.de

Institute for Technical Microbiology, Mannheim University of Applied Sciences, Paul-Wittsack-Str. 10, 68163 Mannheim, Germany

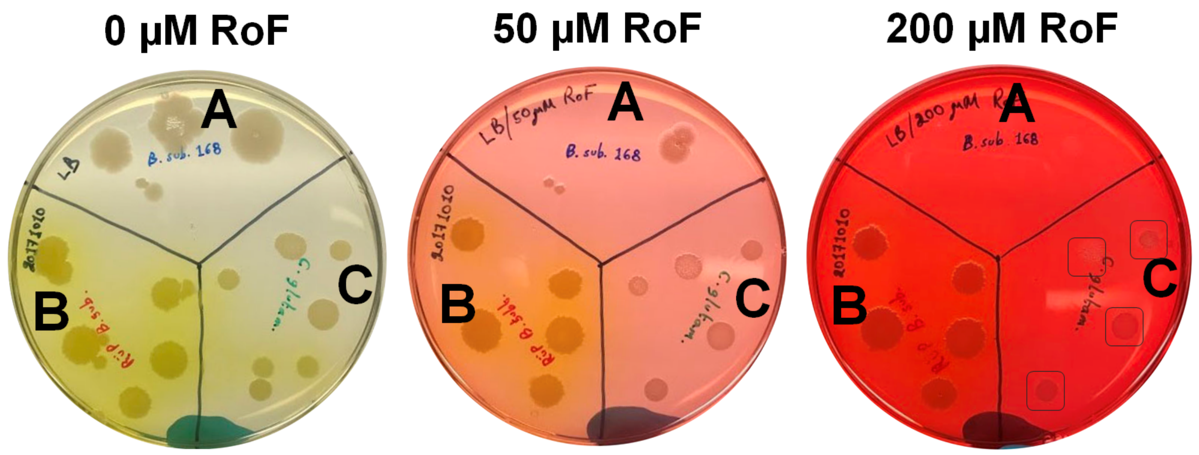

Fig. S2: Roseoflavin (RoF) sensitivity of bacterial strains used in this study. LB agar plates were supplemented with different amounts of roseoflavin (0-200 µM) and inoculated with cells (about 50.000 cells each spot) of *Bacillus subtilis* 168 (A), *Bacillus subtilis* riboflavin overproducing strain ROP (B) and *Corynebacterium glutamicum* MB001DE3 (C) and incubated aerobically for 50 h at 30°C. *B. subtilis* ROP is able to grow in the presence of 200 µM roseoflavin whereas *B. subtilis* 168 does not show growth. *C. glutamicum* MB001DE3 shows strongly reduced growth (see frames). Please note, the yellowish halos in sectors B are a result of riboflavin secretion by ROP.
